# Evolutionary History of the *PER3* Variable Number of Tandem Repeats (VNTR): Idiosyncratic Aspect of Primate Molecular Circadian Clock

**DOI:** 10.1371/journal.pone.0107198

**Published:** 2014-09-15

**Authors:** Flávia Cal Sabino, Amanda Oliveira Ribeiro, Sérgio Tufik, Laila Brito Torres, José Américo Oliveira, Luiz Eugênio Araújo Moraes Mello, Jeferson Souza Cavalcante, Mario Pedrazzoli

**Affiliations:** 1 Department of Psychobiology, Federal University of São Paulo, São Paulo, Brazil; 2 Department of Genetics and Evolutionary Biology, University of São Paulo, São Paulo, Brazil; 3 Evandro Chagas Institute/Primate National Center (IEC-CENP) – Health Surveillance Secretariat, Brazilian Ministry of Health, Ananindeua, Pará, Brazil; 4 Division of Anatomy, Department of Basic Sciences, Araçatuba Dental School, São Paulo State University, Araçatuba, Brazil; 5 Department of Physiology, Federal University of São Paulo, São Paulo, Brazil; 6 Laboratory of Neurochemical Studies, Federal University of Rio Grande do Norte, Natal, Brazil; 7 School of Arts, Science and Humanities, University of São Paulo, São Paulo, Brazil; McGill University, Canada

## Abstract

The *PER3* gene is one of the clock genes, which function in the core mammalian molecular circadian system. A variable number of tandem repeats (VNTR) locus in the 18th exon of this gene has been strongly associated to circadian rhythm phenotypes and sleep organization in humans, but it has not been identified in other mammals except primates. To better understand the evolution and the placement of the *PER3* VNTR in a phylogenetical context, the present study enlarges the investigation about the presence and the structure of this variable region in a large sample of primate species and other mammals. The analysis of the results has revealed that the *PER3* VNTR occurs exclusively in simiiforme primates and that the number of copies of the primitive unit ranges from 2 to 11 across different primate species. Two transposable elements surrounding the 18th exon of *PER3* were found in primates with published genome sequences, including the tarsiiforme *Tarsius syrichta*, which lacks the VNTR. These results suggest that this VNTR may have evolved in a common ancestor of the simiiforme branch and that the evolutionary copy number differentiation of this VNTR may be associated with primate simiiformes sleep and circadian phenotype patterns.

## Introduction

The light/dark cycle that arises from the geospatial relationship between the Earth and the Sun has been shown to be the strongest environmental clue, or *zeitgeber*, for the entrainment of circadian rhythms in many organisms, including human beings [1], [2]. Virtually all physiological processes, such as hormone secretion, sleep, and body temperature which oscillate with circadian periodicity, are regulated by the light/dark signal [3]. The processing of this signal is associated with the expression of a group of genes, collectively known as *clock genes*, which are directly involved in the regulation and maintenance of the circadian rhythms [4], [5]. Due to the fact that variations in these genes, such as polymorphisms and mutations, are associated with aberrant or differential expression of circadian behaviors (reviewed in [6]), the clock genes are strong candidates for natural selection associated with the light/dark cycle signal.

*Period circadian clock 3* (*PER3*) is one of the clock genes engaged in the core mammalian molecular circadian system [7], [8]. In humans, this gene is located on the short arm of chromosome 1 (1p36) [9], and its 18th exon contains a polymorphic variable number of tandem repeats (VNTR) composed of a 54-bp motif that is repeated either four or five times [10]. This length polymorphism in the *PER3* gene has been described in few primate species, such as chimpanzees, gorillas, gibbons, langurs, marmosets, and orangutans, but not in non-primate mammals, such as rats, dogs, and mice [11]. Several reports have shown associations between this VNTR and human circadian rhythm phenotypes, including morning/evening preferences, delayed sleep phase disorder (DSPD), and homeostatic regulation of sleep [10], [12]–[16], highlighting an important link between genetics, the sleep/wake cycle and adaptation to the light/dark cycle.

Due to the importance of the *PER3* VNTR for regulation of sleep and circadian rhythms in humans, it is a matter of interest to investigate the hypothesis that this genomic structure is exclusive to primates. This would allow rationalizing whether this VNTR is associated to some special feature on the primate circadian system. Since, with very few expections, Simiiforme primates are essentially diurnal or cathemeral animals [17], [18] and Prosimians are almost all nocturnal, it would be pertinent to ask whether this VNTR is associated with activity phase allocation during the day or the night. The present study aimed to analyze and compare the *PER3* VNTR regions of several primate species and other non-primate mammals.

## Materials and Methods

### Biological Material from Primate Species

Blood samples were collected from 129 individuals belonging to 13 New World primate species (Table 1) maintained at the *Tufted Capuchin Procreation Center* of Universidade Estadual Paulista (Araçatuba *campus*, Brazil), at the *Zoological Park of São Paulo*, at *Centro Nacional de Primatas* (CENP, Ananindeua, Pará, Brazil), at the *Marmoset Vivarium* of Universidade Federal de São Paulo (São Paulo *campus*), and at *Centro de Primatas* of Universidade Federal do Rio Grande do Norte.

**Table 1:** Primate species analyzed in the present study.

Common name	Species name	Family	Order	IndividualsAnalyzed	GenBankaccession n#	Genome coverage
Grivet	*Chlorocebus aethiops*	Cercopithecidae	Primates	2	KC146097	*
Goeldi’s marmoset	*Callimico goeldii*	Cebidae	Primates	2	KC146101	*
Common marmoset	*Callithrix (Callithrix) jacchus*	Cebidae	Primates	80	KC146106	6X (Mar 2009)
White-headed marmoset	*Callithrix (Callithrix) geoffroyi*	Cebidae	Primates	1	KC146096	*
Red-handed tamarin	*Saguinus midas*	Cebidae	Primates	1	KC146100	*
Emperor tamarin	*Saguinus imperator*	Cebidae	Primates	1	KC146098	*
Brown-mantled tamarin	*Saguinus fuscicollis*	Cebidae	Primates	1	KF408263/KF408264	*
Tufted capuchin	*Cebus apella*	Cebidae	Primates	33	KC146102	*
Common squirrel monkey	*Saimiri sciureus*	Cebidae	Primates	1	KC146099	*
Kuhl’s owl monkey	*Aotus azarae infulatus*	Aotidae	Primates	2	KC146095	*
Black howler	*Alouatta caraya*	Atelidae	Primates	2	KC146105	*
Red-faced spider monkey	*Ateles paniscus*	Atelidae	Primates	2	KC146104	*
Brown woolly monkey	*Lagothrix lagotricha*	Atelidae	Primates	1	KC146103	*

*Genomic assembly information not available.

Classification according to “Wilson & Reeder’s Mammal Species of the World, 3rd Edition”, available at the Taxonomic Browser of the Smithsonian National Museum of Natural History <http://www.vertebrates.si.edu/msw/mswcfapp/msw/index.cfm>.

This study was approved by the Local Committee for Ethics in Research of Universidade Federal de São Paulo (UNIFESP/EPM 0136/05) and includes authorization for sample collections at all animal facilities located inside the universities and within the zoological park. Blood collection concerning wild animals was authorized by the Brazilian Institute of Environment and Renewable Natural Resources (IBAMA) under license #16198. IBAMA, through Deliberation #40/2003 of the Ministry of Environment and the Genetic Heritage Management Council, is the institution responsible for issuing permits for access and delivery of genetic heritage for research activities in the area of biology throughout the Brazilian territory.

All animals were housed under natural conditions of temperature and humidity in a light-dark cycle. The cages were constructed in brick and wire mesh measuring 0.9×2.0×1.8 m, equipped with wood, rope, perches, basket, concrete platforms, cooler box and nest platforms. Water was available *ad libitum*, and food was provided twice a day: in the morning hours between 7∶00 and 9∶00 am, and in the afternoon between 1∶00 and 3∶00 pm. The feed consisted of seasonal fruits and a rich-in-protein preparation that supplemented the diet. Candies were also sporadically offered to animals. At the end of blood sampling the animals were not euthanized. During blood collection, the animals were immobilized by suitable equipment. All animals were in excellent health. After collection the animals received a bounty of candy. The experimental procedures were in accordance with the Guidelines for the Care and Use of Mammals in Neuroscience and Behavioral Research of the National Research Council.

### Extraction, gene amplification, and sequencing

Blood samples were collected into PAXgene Blood RNA vacutainer tubes (PreAnalytiX, Hombrechtikon, Switzerland), and frozen at –20°C. Total RNA or genomic DNA (gDNA) were extracted from the animals’ white blood cells using the PAXgene 96 Blood RNA Kit (QIAGEN, Hilden, Germany). The target cDNA or gDNA regions were amplified by PCR using the primers described by Jenkins and colleagues (5′ AGCAGYTCACCSTTRCAGTT 3′ and 5′ GGYACCTGGTATGTCATGAGAA 3′) [11] or the following pair of primers: 5′ GACTAACAGGTGGGTGGCA 3′ and 5′ CAGAACTTTTTGGGGTGAC 3′. The PCR amplicons were sequenced, and the sequences obtained were submitted to NCBI GenBank (Table 1).

### *In silico* analysis an comparisons of the VNTR region sequence among primate and non-primate species

Multiple alignments of the *PER3* nucleotide sequences obtained were performed using the ClustalW algorithm [19]. The alignments were then visually examined to ascertain the presence of the VNTR and the number of repeats in each primate species. In addition, the PER3 protein sequences from several mammalian species, including prosimians (Table 2), were obtained from the Ensembl Genome Browser Database (http://www.ensembl.org) and were examined for the presence of the target VNTR by aligning the human PER3 protein sequence and the orthologous region from the selected mammals. *In silico* searches for repetitive elements surrounding the VNTR were conducted using the RepeatMasker 3.1.8 online software (http://www.repeatmasker.org).

**Table 2:** Non-simiiforme primates and non-primate mammal species analyzed in the present study.

Common name	Species name	Family	Order	Ensembl Gene ID	Genome coverage
Gray short-tailed opossum	*Monodelphis domestica*	Didelphidae	Didelphimorphia	ENSMODG00000005802	7.33X (Out 2006)
Tammar wallaby	*Macropus (N.) eugenii*	Macropodidae	Diprotodontia	ENSMEUG00000010189	2X (Nov 2009)
Rock hyrax	*Procavia capensis*	Procaviidae	Hyracoidea	ENSPCAG00000004848	2.19 (Jul 2008)
African bush elephant	*Loxodonta africana*	Elephantidae	Proboscidea	ENSLAFG00000000581	7X (Jul 2009)
Nine-banded armadillo	*Dasypus novemcinctus*	Dasypodidae	Cingulata	ENSDNOG00000009963	2X (May 2009)
Hoffmann’s two-toed sloth	*Choloepus hoffmanni*	Megalonychidae	Pilosa	ENSCHOG00000007396	2.05X (Sep 2008)
Northern treeshrew	*Tupaia belangeri*	Tupaiidae	Scandentia	ENSTBEG00000003546	2X (Jul 2006)
Gray mouse lemur	*Microcebus murinus*	Cheirogaleidae	Primates	ENSMICG00000008437	1.93X (Jun 2007)
Northern greater galago	*Otolemur garnettii*	Galagidae	Primates	ENSOGAG00000009800	1.5X (Mar 2011)
Philippine tarsier	*Tarsius syrichta*	Tarsiidae	Primates	ENSTSYG00000007736	1.82X (Jul 2008)
Guinea pig	*Cavia porcellus*	Caviidae	Rodentia	ENSCPOG00000022169	6.79X (Mar 2008)
Ord’s kangaroo rat	*Dipodomys ordii*	Heteromyidae	Rodentia	ENSDORG00000015158	1.85X (Jul 2008)
Thirteen-lined ground squirrel	*Spermophilus (I.)* *tridecemlineatus*	Sciuridae	Rodentia	ENSSTOG00000014007	2X (Nov 2011)
Large flying fox	*Pteropus vampyrus*	Pteropodidae	Chiroptera	ENSPVAG00000005395	2.63X (Jul 2008)
Little brown myotis	*Myotis lucifugus*	Vespertilionidae	Chiroptera	ENSMLUG00000015940	7X (Sep 2010)
Domestic cat	*Felis catus*	Felidae	Carnivora	ENSFCAG00000004371	
Horse	*Equus caballus*	Equidae	Perissodactyla	ENSECAG00000018928	6.79X (Sep 2009)
Bovine	*Bos taurus*	Bovidae	Artiodactyla	ENSBTAG00000014604	
Bottlenose dolphin	*Tursiops truncatus*	Delphinidae	Cetacea	ENSTTRG00000010402	2.59X (Jul 2008)

Classification according to “Wilson & Reeder’s Mammal Species of the World, 3rd Edition”, available at the Taxonomic Browser of the Smithsonian National Museum of Natural History <http://www.vertebrates.si.edu/msw/mswcfapp/msw/index.cfm>.

## Results

**Figure 1:**
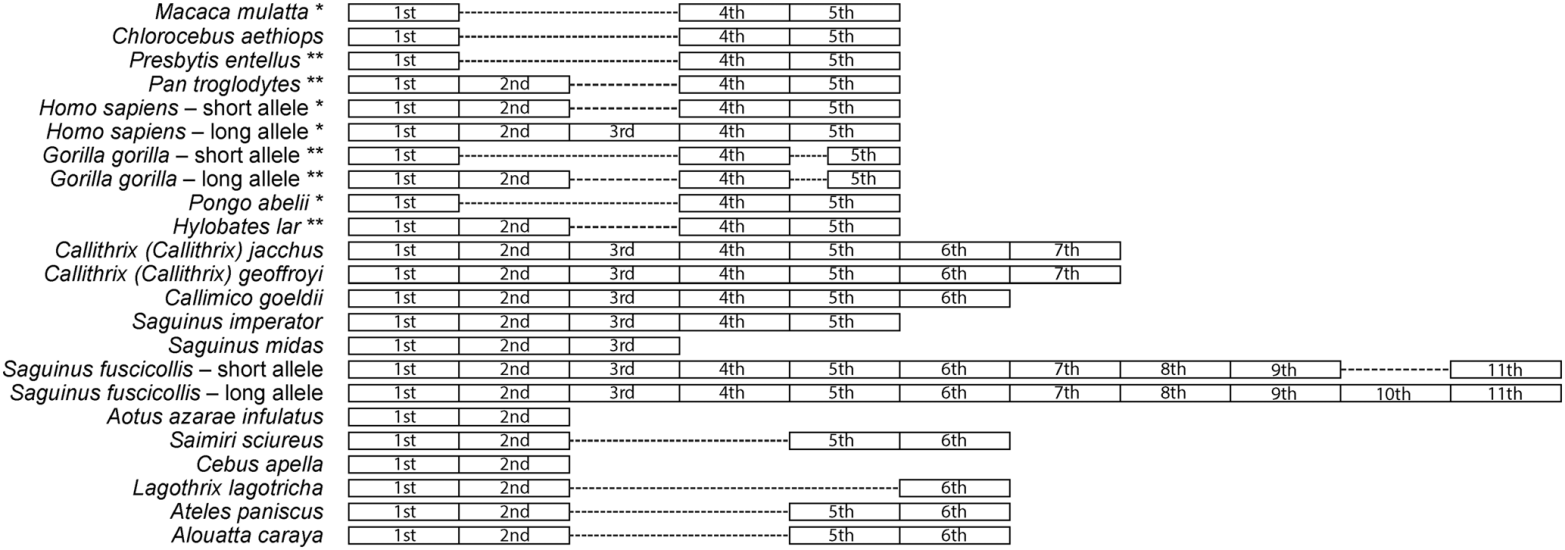
Schematic representation of the alignment of the *PER3* VNTR regions from several primate species. Each rectangle represents one repeat of the VNTR. *Sequences obtained from GenBank (http://www.ncbi.nlm.nih.gov/genbank/). **Sequences obtained from Jenkins *et al*. (2005).

The DNA analysis of the *PER3* VNTR region belonging to the New World primates revealed that all of the studied individuals carried this locus in their genomes, although the number of repeats varied among the different species (Figure 1 and Figure S1). The tufted capuchins and Kuhl’s owl monkeys analyzed had only two repeats. The brown woolly monkey, the two grivets and the red-handed tamarin had three repeats, whereas the two black howlers, the two red-faced spider monkeys and the common squirrel monkey had four. The emperor tamarin had five repeats, the two Goeldi’s marmosets had six, and the white-headed marmoset had seven (Figure 1).

**Figure 2:**
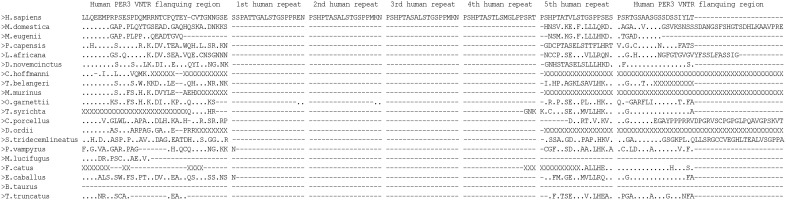
Alignment of the PER3 protein sequences from diverse mammal species, in the region corresponding to the human *PER3* VNTR. The sequences were obtained from the Ensembl Genome Browser Database (http://www.ensembl.org). Dots (.) indicate identity with human sequence. Dashes (–) indicate gaps. X’s indicate unknown or unspecified aminoacids.

The analysis of the PER3 protein sequences from several mammalian species revealed that the amino acid sequence in this region differed both in size and sequence among species (Figure 2). No full repeats of the 18 amino acids of this VNTR were found among the prosimians and non-primate mammals and, although some of the non-primate mammalian sequences aligned with one of the repeat units of the human VNTR, the alignment score was low. Only a few species exhibited scattered amino acid identity to the amino acids present in a human unit of the VNTR sequence. Thus, the non-primate mammals examined in the present study had neither repeats of this VNTR nor a single sequence identical to a human unit of the 18 amino acids. We would like to emphasize that the *in* *silico* analysis of the PER3 VNTR region of three species of prosimian primates (*Tarsius syrichta*, *Otolemur garnettii*, and *Microcebus murinus*) also revealed an absence of repeats (Figure 2).

**Figure 3:**
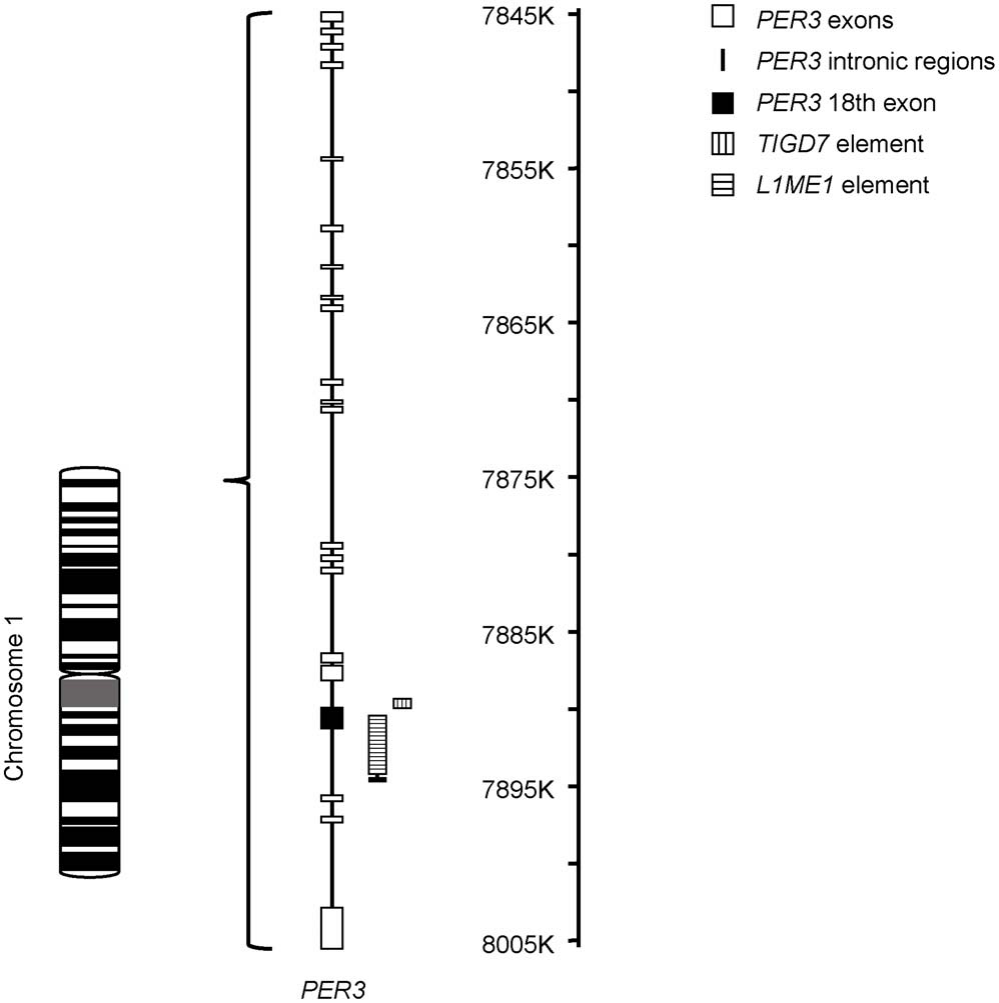
Representation of the *PER3* human gene and the repetitive sequences associated to it. The black square represents the 18^th^ exon of *PER3*, where the VNTR is placed. Adapted from NCBI Map Viewer (http://www.ncbi.nlm.nih.gov/projects/mapview).

In addition, the bioinformatics analysis showed that the *PER3* VNTR is surrounded by transposable elements. The DNA transposon *tigger transposable element derived 7* (*TIGD7*) is inserted upstream of the VNTR at the end of the intron and a few nucleotides before the beginning of the first repeat. Downstream of the VNTR, a LINE-1 element (*L1ME1*) is inserted near the end of the last repeat (Figure 3). Interestingly enough, although the prosimian *Tarsius syrichta* contains these two transposable elements in this region of the gene, such species does not have the VNTR. Moreover, the non-primate mammals analyzed did not exhibit transposable elements nor VNTR.

## Discussion

The present study demonstrates that the VNTR in the *PER3* gene is a genomic structure present in all Simiiforme primates studied, although the number of repeats is different, depending on the species. We have found that the number of repeats range from 2 to 11 (Figure 1). Observation of these results indicates that other similar primates, not studied here, may also carry the VNTR, thus indicating that this structure is present exclusively in primates [11]. Platyrrhini (New World monkeys) exhibits greater variability. For example, the species belonging to genus *Aotus* (the only established nocturnal simiiforme primate) exhibit only two repeats, whereas genus *Saguinus* carries up to 11 repeats.

*In silico* analysis of the flanking regions concerning the 18th exon of *PER3* revealed that both introns adjacent to the VNTR contain transposable elements (Figure 3). The sequences of the two transposable elements surrounding the *PER3* VNTR region are highly similar in all the primates analyzed, including the prosimian *Tarsius syrichta*, and are absent in non-primate mammals. This finding suggests that these elements were present in the common ancestor of Tarsiiformes and Simiiformes, which probably lived during the Eocene Period [20], [21], and that the insertion of the transposable elements occurred prior to the appearance of the *PER3* VNTR. Moreover, the absence of these sequences in non-primate mammals indicates that their insertion into these *loci* was a primate lineage-specific event.

Transposable elements are known to produce genomic instability, rearrangements, genetic innovation [22]–[28], and are associated with the generation of tandem repeats [29]. Therefore, one might tentatively propose that the primitive insertion of these transposable elements in the primate lineage caused the emergence of the VNTR in the *PER3* gene in this Order.

**Figure 4:**
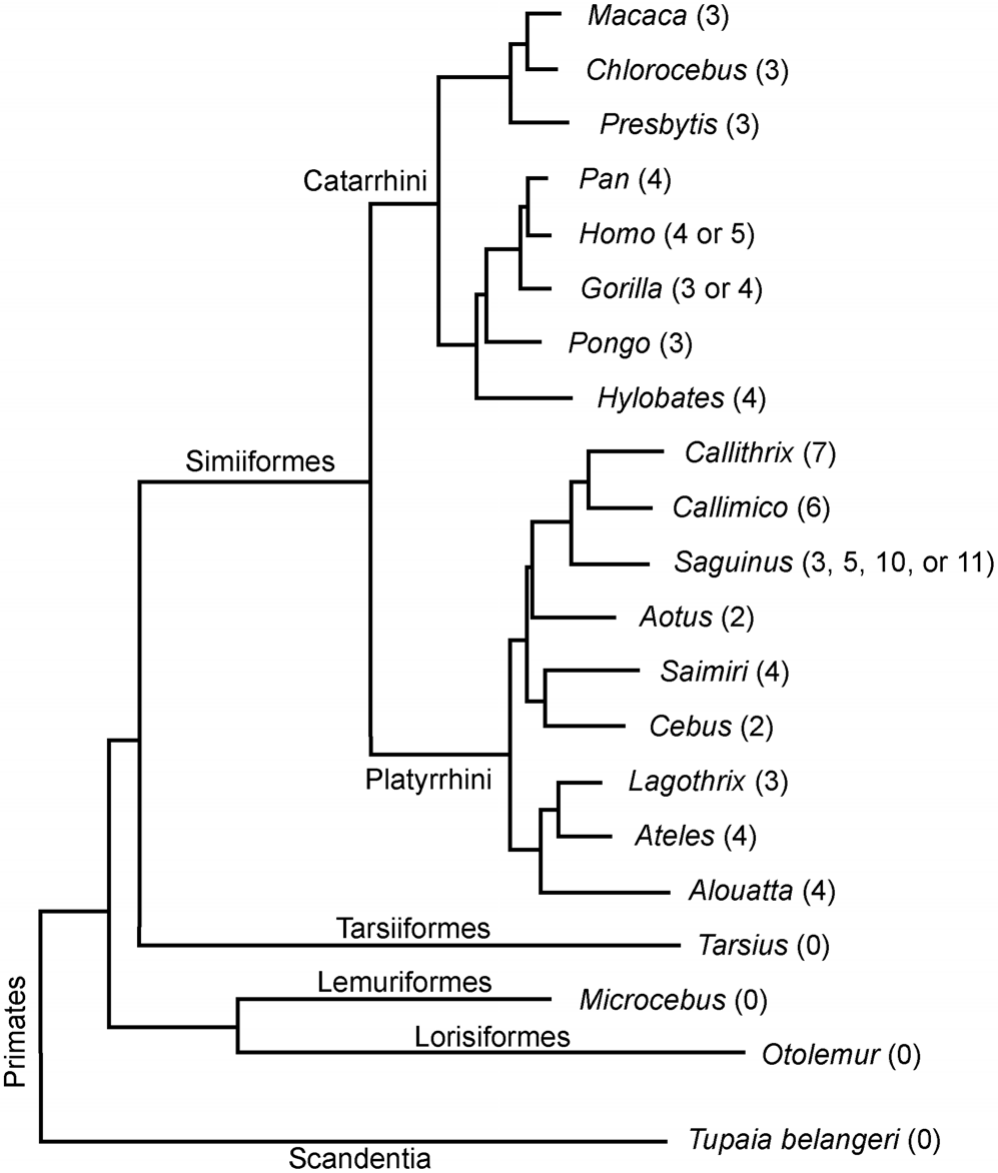
Evolutionary relationships among the analyzed primate genera, based on the phylogeny proposed by Perelman *et al*. (2011). Between brackets, the number of repetitions comprising the *PER3* VNTR for each genus.

The results of the present study, combined with data generated in a previous report [11], enable a reasonable reconstruction of the primate PER3 VNTR evolution (Figure 4). The interpretation of these results largely follows the explanation provided by Jenkins and colleagues [11] who suggest that the VNTR was derived from a single ancestral unit in the common ancestor of primates, carnivores, and rodents. However, data from the additional species analyzed herein and the additional finding of transposable elements flanking the VNTR region suggest that, in fact, the VNTR derived from a single copy that was present in the common ancestor of Simiiformes, and that a duplication event most likely occurred in primates before Catarrhini and Platyrrhini diverged, approximately 43.5 million years ago [20], [21]. The repeat thus created was then subject to a variable number of posterior duplications and, perhaps, deletions.

Tandem repeats exhibit mutation rates much higher than those of other genomic regions. When they are located inside genes and regulatory regions, tandem repeats may influence the properties or functions of these genes, such as binding sites, chromatin structure, and, ultimately, gene transcription [30]. A VNTR that is located within a coding region may affect the stability and/or activity of the gene product [31], [32]. Although molecular mechanisms underlying gene functional changes mediated by tandem repeats are generally poorly understood, tandem repeat domains in proteins are usually involved in protein-protein interactions and fine-tuning protein conformation [33]. Nevertheless, it is unlikely that variations in tandem repeats produce completely new characteristics; rather, they allow the fine-tuning of specific phenotypes, including those related to behavior, physiology, and morphology, due to the wide range of allelic variation [34], thus switching from most night to most day activity patterns; in the case of primates, would fit with the fine-tuning phenotype proposal. A clear documented case for this phenomenon, for instance, is that variations in the number of repeats of a hexanucleotide in exon 5 of the *Period* gene influence the mechanism of temperature compensation in *Drosophila melanogaster* [35], [36].

The uniqueness of the *PER3* VNTR in primates and its strong association with circadian phenotypes and homeostatic sleep regulation in humans indicate that this VNTR is associated with a special feature of the primate circadian system or sleep. When compared with other mammalian species, Simiiforme primates exhibit such special features as consolidated monophasic or biphasic sleep [37] and a diurnal activity pattern. The owl monkeys from the genus *Aotus* are an exception between the Simiiformes [38], and the Prosimians, more primitive primates, are mostly nocturnal. Interestingly, the present study showed that the analyzed *Aotus* species possesses only two repeats of the *PER3* VNTR, and the Prosimian species, none.

It is generally assumed that the ancestor of mammals - and of primates in particular - was a nocturnal creature [39]. Although most of the recently evolved mammals are nocturnal, Simiiformes definitively are not. Repetitive sequences may provide a high degree of evolutionary flexibility, allowing adaptive accommodations at a minimal cost to the genetic function. Thus, perhaps the *PER3* VNTR is involved as a part of the mechanism that provides evolutionary flexibility that allowed the occupation of different portions of the light/dark niche - in the case of Simiiformes, the lightened portion of the cycle.

While some genomes used for the sequence assembling have high sequencing depths, such as the genomes of human, chimp, mouse and dog, others, such as the gray mouse lemur genome, present only low levels of sequence coverage. Although a complete coverage of the genomes used for the study is obviously preferable, the usage of lower-redundancy genomes may be the only alternative when investigating non-laboratory model species [40]. While low-percent coverage brings some limitations for sequence analysis, utilization of only deeper-covered genomes would substantially reduce the number of targets, crashing the possibility of a phylogenetic approach.

In summary, the present study demonstrates that the *PER3* VNTR is a genomic structure found exclusively in the primate *PER3*, a gene found only among vertebrates. This VNTR is flanked by two transposable elements that are phylogenetically older than the VNTR itself and that may have been involved in the emergence of this structure. The present findings also show that the nocturnal *Tarsius* does not possess this VNTR and that primates of the genus *Aotus*, which are nocturnal, have the smallest number of copies of the VNTR of almost all the primates analyzed.

## Supporting Information

Figure S1 **Alignment of the** ***PER3*** **VNTR regions from several primate species.** Each repetition is represented in a different color. *Sequences obtained from GenBank (http://www.ncbi.nlm.nih.gov/genbank/). **Sequences obtained from Jenkins *et al*. (2005).
(TIF)
