# Supplementary figures and images for: Evolutionary History of the PER3 Variable Number of Tandem Repeats (VNTR): Idiosyncratic Aspect of Primate Molecular Circadian Clock
Source: PLoS One. 2014 Sep 15;9(9):e107198. doi: 10.1371/journal.pone.0107198 (PMC4164614; doi:10.1371/journal.pone.0107198)

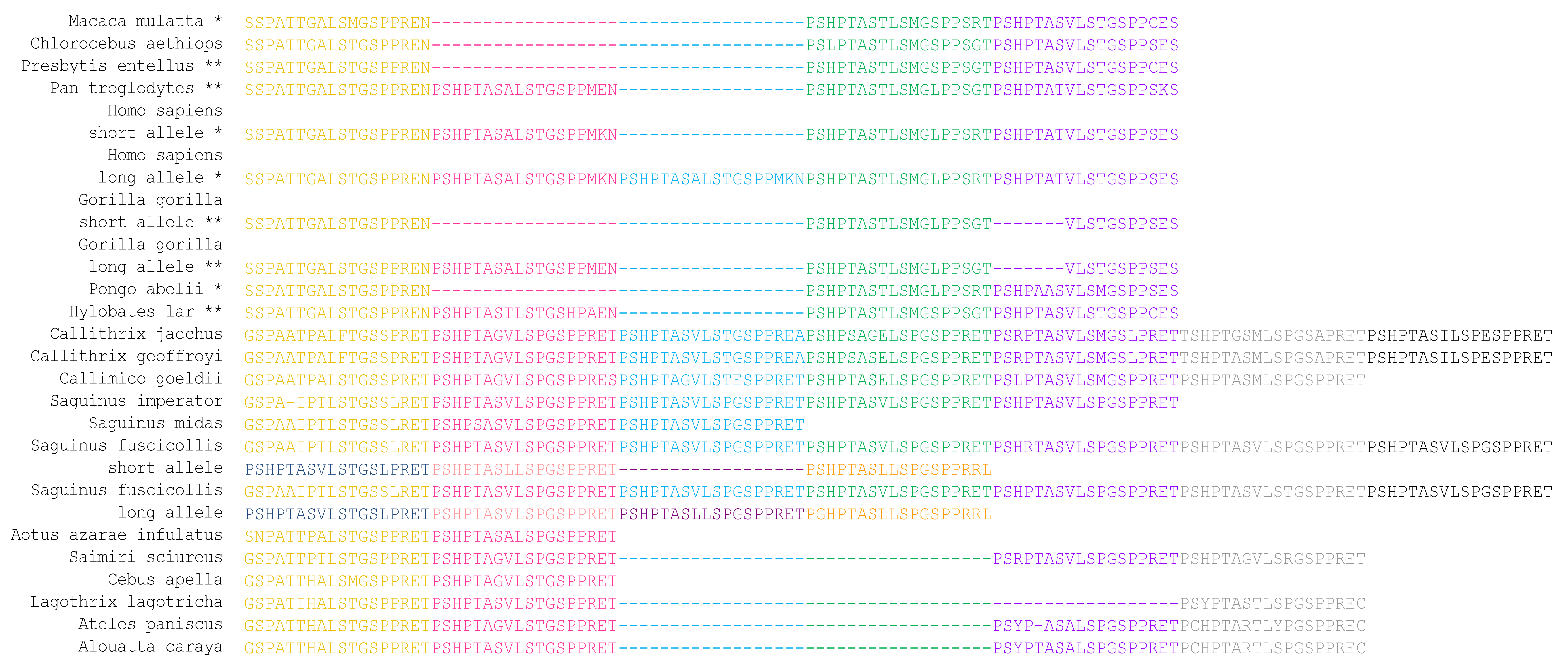

Supplement: Figure S1 — Alignment of the PER3 VNTR regions from several primate species. Each repetition is represented in a different color. *Sequences obtained from GenBank (http://www.ncbi.nlm.nih.gov/genbank/). **Sequences obtained from Jenkins et al. (2005). (TIF) [file pone.0107198.s001.tif]
